# Differences in length of stay and discharge destination among patients with substance use disorders: The effect of Substance Use Intervention Team (SUIT) consultation service

**DOI:** 10.1371/journal.pone.0239761

**Published:** 2020-10-09

**Authors:** Hale M. Thompson, Walter Faig, Nicole A. VanKim, Brihat Sharma, Majid Afshar, Niranjan S. Karnik

**Affiliations:** 1 Department of Psychiatry & Behavioral Sciences, Section of Community Behavioral Health, Rush University Medical Center, Chicago, IL, United States of America; 2 Department of Biostatistics and Epidemiology, School of Public Health and Health Sciences, University of Massachusetts Amherst, Amherst, MA, United States of America; 3 Division of Pulmonary and Critical Care, Department of Public Health Sciences, Center for Health Outcomes & Informatics Research, Loyola University, Maywood, IL, United States of America; Azienda Ospedaliero Universitaria Careggi, ITALY

## Abstract

**Background:**

Addiction medicine consultation services (ACS) may improve outcomes of hospitalized patients with substance use disorders (SUD). Our aim was to examine the difference in length of stay and the hazard ratio for a routine hospital discharge between SUD patients receiving and not receiving ACS.

**Methods:**

Structured EHR data from 2018 of 1,900 adult patients with a SUD-related diagnostic code at an urban academic health center were examined among 35,541 total encounters. Cox proportional hazards regression models were fit using a cause-specific approach to examine differences in hospital outcome (i.e., routine discharge, leaving against medical advice, in-hospital death, or transfer to another level of care). Models were adjusted for age, sex, race, ethnicity, insurance status, and comorbidities.

**Results:**

Length of stay was shorter among encounters with a SUD that received a SUIT consultation versus those admissions that did not receive one (5.77 v. 6.54 days, p<0.01). In adjusted analyses, admissions that received a SUIT consultation had a higher hazard of a routine discharge [hazard ratio (95% confidence interval): 1.16 (1.03–1.30)] compared to those not receiving a SUIT consultation.

**Conclusions:**

The SUIT consultation service was associated with a reduced length of stay and an increased hazard of a routine discharge. The SUIT model may serve as a benchmark and inform other health systems attempting to improve outcomes in SUD patient cohorts.

## Introduction

Substance misuse continues to expand across the United States with alcohol and opioid misuse driving this epidemic and related hospitalizations [1–3]. Individuals with substance use disorders (SUD) tend to have greater frequencies of hospitalizations, longer lengths of stay (LOS), and more unplanned readmissions compared to the general population of hospitalized patients [1, 4, 5]. Although the Affordable Care Act expanded funding for treatment and service delivery [6, 7], the ever-expanding opioid epidemic has driven the emergence of addiction consultation services and systems-level interventions in the treatment of SUD [8]. From 2002–2012, opioid-related hospitalizations nearly doubled and hospital charges quadrupled nationally for SUD-related hospitalizations [9]. However, substantial heterogeneity in healthcare costs exist regionally due to differing opioid supply and demand drivers [9], as well as differing rates of Medicaid expansion adoption [1, 10]. One study [11] found that SUD-related care accounted for 20% of Medicaid general hospital stays. Further, many hospitalizations for a physical health problem, including HIV, hepatitis C, cirrhosis, endocarditis, malignancies, stroke, diabetes, heart disease, and asthma, have a co-occurring SUD [11, 12].

Effective treatment pathways for SUD are needed to reduce the strain on healthcare systems and the costs of care associated with this increasing condition among hospitalized patients [13]. Psychiatric consult services have effectively reduced average LOS [14], and the more recent emergence of addiction medicine consult services (ACS) have shown striking improvements in patient and hospital outcomes [15–18]. An ACS can effectively integrate medication treatment for opioid use disorder (OUD) and alcohol use disorder (AUD) which has been shown to reduce misuse, related morbidity and mortality, and substantially reduce costs associated with long hospital stays; the medication treats cravings and withdrawal symptoms and allows patients, with infections related to misuse, for example, to stabilize at home or in a skilled nursing facility rather than an extended hospitalization [2, 19].

The Substance Use Intervention Team (SUIT) service was established as a systems-level approach to identify, treat, and link to outpatient care, while reducing LOS, those hospitalized patients with SUD. SUIT combines a universal screening model, ACS for inpatient intervention, and an outpatient addiction medicine discharge clinic. SUIT patients are typically discharged home or to a skilled nursing facility, if they need time to stabilize comorbid conditions, and linked directly to the discharge clinic or another outpatient treatment service depending upon insurance coverage and eligibility. Preliminary data from the first five months of the SUIT service demonstrated nearly a full day shorter LOS for patients who received a SUIT consultation compared to patients who did not; however, this difference was not statistically significant, potentially due to small sample sizes (5.91 v. 6.73 days, p = 0.07) [20].

### Objectives

The SUIT program as an example of integrated ACS serves many important functions for effective treatment of substance misuse during hospitalization including patient experience [21–24], clinician experience [25], and care quality [18, 26]. The present analysis focuses on the SUIT program’s potential impact on key cost drivers for the hospital system, namely LOS and routine discharge to home, and aims to: (1) identify differences in LOS over a 12-month period and (2) estimate the hazard of a hospitalization ending in a routine discharge home versus the alternative, competing events of leaving against medical advice (AMA), in-hospital death, or a transfer to another level of care. These objectives estimate whether the SUIT program is effective as a harm reduction approach across any SUD diagnosis in improving these hospital outcomes and, further, generate hypotheses about outcome differences that may be associated with treatment, care quality, or social determinants of health.

## Material and methods

### Environment and the SUIT intervention

The study sample consists of a cohort of patients admitted to the inpatient units of an urban, academic health center between January 1, 2018 and December 31, 2018. The health system is located in Illinois, a Medicaid-expansion state, and is adjacent to urban communities with the highest rates of heroin overdose and lowest socioeconomic position in the City of Chicago [27]. To help mitigate the rising inpatient healthcare utilization and expenditures, frequently observed in patients with SUD, our health system implemented the SUIT program as an extension of a Substance Abuse and Mental Health Services Administration (SAMHSA) Screening Brief Interventional and Referral to Treatment (SBIRT) training grant. Although the patient may decline participation at any point of the screening and treatment cascade, the SBIRT begins with admission nurses asking a two-question drug and alcohol universal pre-screen. Alcohol Use Disorders Identification Test (AUDIT) or Drug Abuse Screen Test (DAST) screens are then completed for positive pre-screens by inpatient social workers. These scores are used to risk-stratify patient substance use [28]. Moderate-risk patients receive brief education (DAST: 1, AUDIT: 1–4) or brief intervention (DAST: 2, AUDIT: 5–12) such as psychosocial education and/or motivational interviewing. DAST scores greater than 3 indicate harmful or severe use, and trigger SUIT consultation. AUDIT scores greater than 13 indicate harmful or severe use, and prompt SUIT consultation. SUIT consultation typically includes medication treatment initiation, motivational interviewing, harm reduction education (e.g., Naloxone training), discharge planning, and linkages to resources, such as the SUIT discharge clinic. In combination with medication, the motivational interviewing and warm handoff aim to reduce length of stay and release patients home for outpatient treatment. The SUIT staffing consists of physicians, psychiatric nurse practitioners, a clinical pharmacist, licensed clinical social workers, a nurse, and a medical assistant. The SUIT program and floor social workers, trained to conduct the secondary screening, are available Monday-Friday 9am-5pm. The team rounds on patients daily and continues to see them if they are linked to the discharge clinic.

In practice, many SUIT consultations are ordered by the primary medical team due to the severity of the admitted patient’s presentation, thereby circumventing our universal screening pathway with assessment using the AUDIT and DAST. In this way, the universal screening runs alongside, rather than fully integrated, with the SUIT consultation process. Similarly, for SUIT patients discharged home, the EHR does not capture where those patients may have been linked to outpatient treatment.

### Conceptual model

The SUIT program is based on principles of harm reduction [22, 29]. The intervention accounts for gradations of misuse and the incremental stages of change with respect to reducing misuse by using a risk-stratified screening process and by consenting patients for education and treatment. SUIT providers treat patients non-judgmentally and as autonomous, accountable decisionmakers and recognize that pervasive stigma and lack of social supports create barriers to progress and change.

### Cohort sample and eligibility criteria

Patients were eligible for inclusion in the cohort if they were 18 years or older at the time of admission and their hospitalization included any *International Classification of Diseases*, *Tenth Revision*, *Clinical Modification (*ICD-10-CM) diagnostic codes for SUD during their stay (F10-F19 prefixes with the exception of F17 for nicotine dependence). In 2018, there were 2,518 hospital encounters and 1,900 unique patients with an eligible ICD-10-CM code. Three hospitalizations were omitted due to missing data. Eighty-seven percent of the 2018 encounters received the 2-question drug and alcohol screening. Of those screened, 47% were positive for alcohol misuse and 39% were positive for drug misuse in the last year. AUDIT and DAST scores were entered for 49% of eligible encounters, and 61% and 67% of the AUDIT and DAST scores, respectively, met or exceeded the threshold for a consult. Because 50% of AUDIT and DAST scores are missing, and because patients who screen positive do decline the consult service, AUDIT and DAST scores are not eligibility criteria. Of note, patients without AUDIT or DAST scores who are treated by the SUIT team upon admission due to the severity of their presentation, may later choose not to continue with outpatient treatment; they are, however, counted as patients who received a SUIT consultation. In total, 31.4% (n = 597) of unique patients with a SUD received one or more SUIT consultations in 2018.

### Statistical analysis

For the first aim, the primary outcome was LOS in days, comparing SUD patients who received a SUIT consultation to those not receiving a SUIT consultation. A t-test was performed to determine differences in LOS at both encounter-level and patient-level and then stratified by discharge status. Chi-square tests and tests of proportions were conducted across demographics and covariates collected in the EHR to identify differences between patients who received a SUIT consultation and those who did not.

The second aim examined hospital discharge status as the outcome and consisted of a multivariable competing risk analysis tested at the encounter level. Cox proportional hazards regression models were fit using a cause-specific approach due to its treatment of individuals experiencing competing events [30]. The cause-specific model estimates the probability that the SUIT consultation potentially affects the hazard at which patients who are event-free are discharged home–known as routine discharge. Although the cause-specific model does not estimate the effect on the cumulative incidence function [30, 31], it enables a nuanced exploration of the SUIT consultation’s association with discharge status while adjusting for salient covariates [32–34]. Length of stay was defined as the time from hospital admission to discharge from the hospital, and LOS was right-censored at 28 days, more than four times the average length of stay for a SUD encounter in 2018. Competing events were also right-censored at the time the competing event occurred. Sensitivity analyses were performed to examine each type of discharge status as the primary outcome of interest. Specifically, the other three discharge categories are: transfer to another level of care, discharge AMA, and in-hospital death. In general, transfers span eight categories of care such as psychiatric facilities to hospice; for SUD patients, 90% of transfers are to a skilled nursing facility or long-term care facility to stabilize until independent living is feasible.

Covariate selection was performed *a priori* based on available EHR data and a literature review of risk factors [18, 35, 36]. The covariates included in the adjusted model include: age (continuous), sex (female referent), race (Black referent), ethnicity (Hispanic referent), and payor/insurance status (private insurance referent), and Elixhauser score (continuous). The Elixhauser mortality score uses diagnostic codes in administrative data to account for major comorbidities including mental health conditions and substance use disorder diagnoses [37]. All analyses had a p<0.05 level of significance and were conducted using R version 3.5.1 (R, Boston, MA). The study protocol was approved by the Rush University Medical Center Institutional Review Board. Consent was not obtained as retrospective data were deidentified and analyzed anonymously.

## Results

### Sample demographic and utilization characteristics

Table 1 presents descriptive characteristics of patients comparing those who had a SUIT consultation to those who did not receive a SUIT consultation. The mean age of unique patients was 50 years (SD = 14.70; range: 18–92 years), and the mean age of patients who received a SUIT consultation was lower than those who did not (48.43 v. 50.71 years, p<0.001). There were no significant differences across sex, race, and ethnicity. However, there were differences across payor status and disorder diagnosis (p<0.001). Among those receiving a SUIT consultation, more patients had Medicaid (52.43% vs. 40.68%) or no health insurance (7.71% vs. 3.61%), and more carried an OUD diagnosis (31.32% vs. 23.56%) or both an OUD and AUD (14.74% vs. 6.29%). For patients without a consult, a greater proportion had Medicare (17.25% vs. 27.32%), and more carried an AUD diagnosis (46.90% vs. 55.10%) or another SUD diagnosis (7.04% vs. 15.04%) besides AUD or OUD. At the encounter level, discharge status differed between those with a SUIT consultation compared to those without one (p<0.001); routine discharges were similar between groups (58% vs. 57%), but patients without a SUIT consultation had higher proportions of transfers (30% vs. 36%) and deaths (1.60% vs. 3.30%) and a lower proportion who left AMA (11% vs. 3%) compared to patients receiving a SUIT consultation.

**Table 1:** Descriptive characteristics of unique patients (N = 1,900) and discharge status for total admissions (N = 2,515) in 2018 for any SUD diagnoses.

Unique Patient Characteristics	N = 1,900						
	Had SUIT Consultation (n = 597)		Had no SUIT Consultation (n = 1,303)				p-value^a^
Age [years; mean (SD)]	48.43 (13.04)		48.43 (13.04)				<0.001
	n	%	n	%		χ^2^ (df)	p-value^b^
Sex						0.26 (1)	0.61
Female	204	34.17%	461	35.38%			
Male	393	65.83%	842	64.62%			
Race						2.15 (3)	0.54
Black	278	46.57%	566	43.44%			
White	219	36.68%	520	39.91%			
Other	86	14.41%	190	14.58%			
Declined/N/A	14	2.35%	27	2.07%			
Ethnicity						4.70 (2)	0.10
Hispanic/Latinx	94	8.57%	181	13.89%			
Non-Hispanic/non-Latinx	498	91.43%	1,095	84.04%			
Refuse/unknown	5	0.84%	27	2.07%			
Payor						49.13 (3)	<0.001
Private	135	22.61%	370	28.40%			
Medicaid	313	52.43%	530	40.68%			
Medicare	103	17.25%	356	27.32%			
Uninsured	46	7.71%	47	3.61%			
Disorder Diagnosis						68.34 (3)	<0.001
Alcohol Use Disorder	280	46.90%	718	55.10%			
Opioid Use Disorder	187	31.32%	307	23.56%			
AUD & OUD	88	14.74%	82	6.29%			
Other SUD	42	7.04%	196	15.04%			
Utilization Characteristics^c^	N = 2,515						
	Had SUIT Consultation (n = 743)		Had no SUIT Consultation (n = 1,772)				
	n	%	n		%	χ^2^ (df)	p-value
Discharge Status						75.04 (3)	<0.001
Routine Discharge Home	429	58.00%	1,016	57.00%			
Transfer to Other Level of Care	221	30.00%	645	36.00%			
In-Hospital Death	12	1.62%	59	3.33%			
Left AMA	81	11.00%	52	3.00%			
Elixhauser Score [mean(SD)]	7.81 (3.75)		7.42 (3.84)				0.02

SUD, Substance Use Disorder; SD, Standard Deviation; SUIT, Substance Use Intervention Team; AUD, Alcohol Use Disorder; OUD, Opioid Use Disorder; AMA, Against Medical Advice.

^a^ p-value calculated from t-test.

^b^ p-value calculated from χ^2^ test.

^c^ Due to multiple admissions per year for some patients the sample size increases.

In 2018, the overall average LOS for all SUD hospitalizations was 6.31 days, and a statistically significant 0.77-day difference was identified between SUD hospitalizations that included a SUIT consultation and those that did not (5.77 v. 6.54 days, p<0.01). At the patient level, the difference was 1.71 days (6.40 v. 8.11 days, p<0.001). Table 2 presents average LOS at the encounter level, stratified by discharge status. Routine discharge reflects the only significant difference in LOS by discharge status (3.99 vs. 4.57 days, p<0.01).

**Table 2:** Average length of stay across types of discharge from hospital stay in 2018.

	Routine Discharge Home	Transfer to Another Level of Care	In-Hospital Death	Left AMA	TOTAL
Length of Stay [days; (n)]	4.40 (1,445)	9.79 (866)	8.99 (71)	2.33 (133)	6.31 (2,515)
SUIT Consultation	3.99 (429)**	9.89 (221)	7.44 (59)	2.21 (52)	5.77** (743)
No SUIT Consultation	4.57 (1,016)	9.77 (645)	16.58 (12)	2.40 (81)	6.54 (1,772)

* p < 0.05

** p < 0.01

*** p < 0.001; comparing had a SUIT consultation to had no SUIT consultation.

### Cox regression analyses and hazard ratios

In the cause-specific competing events model, the proportional hazards assumptions were satisfied according to a test of independence between Schoenfeld residuals and time. SUIT consultation (Table 3) was associated with the event of interest, routine discharge; across LOS, SUD patients who received a SUIT consultation had a statistically significant, higher hazard of routine discharge compared to patients who did not receive the SUIT consultation throughout the follow-up period [hazard ratio (HR) 95% confidence interval (CI): 1.16 (1.03–1.30)]. Further, male [1.13 (1.01–1.26)], and uninsured SUD patients [1.46 (1.16–1.85)] had a higher hazard for routine discharge compared to female SUD patients and patients with private insurance, respectively. Covariates associated with a SUD patient’s lower hazard for routine discharge were age [0.99 (0.98–0.99)], other race [0.74 (0.61–0.89)], white race [0.80 (0.71–0.90)], non-Hispanic ethnicity [0.75 (0.63–0.88)], other ethnicity [0.44 (0.25–0.77)], and each incremental 1-point increase in Elixhauser score [0.87 (0.85–0.88)].

**Table 3:** Cause-specific competing risk model with Cox proportional hazard ratios for routine discharge home among hospitalized SUD patients in 2018 (N = 2,515).

	Routine Discharge Home (n_events_ = 1,438) vs. any other discharge		
	HR	95% CI	p-value
Had SUIT consultation	1.16	(1.03–1.30)	0.013
Age	0.99	(0.98–0.99)	<0.001
Patient Sex			
Female (ref)			
Male	1.13	(1.00–1.26)	0.034
Race			
Black (ref)			
White	0.80	(0.71–0.90)	<0.001
Other/NR	0.74	(0.61–0.89)	0.001
Ethnicity			
Hispanic (ref)			
Not Hispanic or Latino	0.75	(0.63–0.88)	<0.001
Other/NR	0.44	(0.25–0.77)	0.004
Payor			
Private (ref)			
Medicaid	0.93	(0.82–1.05)	0.253
Medicare	0.88	(0.75–1.05)	0.157
Uninsured	1.46	(1.16–1.85)	0.001
Elixhauser Score	0.87	(0.85–0.88)	<0.001

SUD: Substance Use Disorder; HR, Hazard Ratio; CI, Confidence Interval, SUIT, Substance Use Intervention Team; NR, Not Reported.

In sensitivity analyses, SUIT consultation was not associated with a transfer to another level of care, but it was associated with a lower hazard of in-hospital death [0.50 (0.25–0.99)] and a higher hazard of leaving AMA [3.40 (2.39–4.85)] (Table 4). We conducted the competing events survival analysis and sensitivity analyses at the patient-level in order to assess the effect of readmissions on the hazard ratio estimates. Examination of unique patients reduced the sample to n = 1,900 and resulted in a nearly identical pattern to the encounter-level analyses with one notable difference. Hazard ratio estimates for having a SUIT consultation in cause-specific models increased for routine discharge (2.95 [1.91–4.56]), though remained not statistically significant for transfer to another level of care (1.08 [0.97–1.20]). Notably, the hazard for leaving AMA was lower among those receiving a SUIT consultation in this reduced sample (0.41 [0.18–0.91]). The sample was too small to compute a reliable hazard ratio for in-hospital death.

**Table 4:** Cause-specific competing risk model with Cox proportional hazard ratios for each discharge type among hospitalized SUD patients in 2018 (N = 2,515).

	Transfer to Another Level of Care (n_events_ = 835)		In-Hospital Death (n_events_ = 67)		Left AMA (n_events_ = 133)	
	HR	95% CI	HR	95% CI	HR	95% CI
Had SUIT consultation	1.07	(0.91–1.25)	0.50	(0.25–0.99)*	3.40	(2.39–4.85)***
Age	1.02	(1.01–1.03)***	1.01	(0.99–1.03)	0.98	(0.96–0.99)**
Patient Sex						
Female (ref)						
Male	0.95	(0.83–1.10)	1.33	(0.78–2.28)	2.12	(1.42–3.19)***
Race						
Black (ref)						
White	0.96	(0.65–1.09)	0.98	(0.34–1.12)	1.03	(0.70–1.51)
Other/NR	0.84	(0.83–1.13)	0.61	(0.43–2.24)	0.62	(0.30–1.29)
Ethnicity						
Hispanic (ref)						
Not Hispanic or Latino	0.92	(0.74–2.28)	0.85	(0.36–2.03)	1.65	(0.88–3.11)
Other/NR	1.29	(0.71–1.19)	4.51	(1.50–13.54)**	NE	
Payor						
Private (ref)						
Medicaid	1.19	(0.99–1.42)	1.64	(0.83–3.23)	3.19	(1.83–5.55)***
Medicare	1.22	(1.00–1.50)*	1.57	(0.72–3.42)	1.63	(0.77–3.44)
Uninsured	0.61	(0.32–1.16)	1.37	(0.30–6.36)	2.60	(1.08–6.27)*
Elixhauser Score	0.99	(0.97–1.01)	0.97	(0.91–1.04)	0.98	(0.93–1.03)

HR, Hazard Ratio; CI, Confidence Interval, SUIT, Substance Use Intervention Team; NR, Not Reported; NE, Not Estimated.

* p < 0.05

** p < 0.01

*** p < 0.001; compared to any other discharge.

## Discussion

In 2018, the first year of the SUIT service, patients who misuse substances and received a SUIT consultation had a lower average LOS compared to SUD patients who did not receive a consultation. When stratified by discharge status, this shorter LOS was associated with routine discharge. The cause-specific competing event model highlights this association; the consult service is associated with a higher hazard ratio for routine discharge, suggesting that the SUIT service may help mitigate the utilization burden that SUD-related illnesses have on patients, payors, and the academic health center. A shorter LOS among those SUIT patients discharged home is likely tied to four aspects of the SUIT program: 1) the primary medical team’s awareness of and relationship with the SUIT team whereby the consult is ordered soon after an SUD patient’s admission, particularly those patients with severe OUD-related illness, 2) the effectiveness of medication treatment for relief of symptoms related to OUD and AUD, 3) warm handoffs to the SUIT discharge clinic or other outpatient treatment options, and 4) that SUIT operates in a Medicaid-expansion state and covers medication treatment for substance use disorders. From a harm reduction perspective, a reduced LOS for SUIT patients represents reduced costs of hospitalization along with an incremental and pragmatic improvement in treatment pathways, that account for the individualism of each patient and the autonomy and shared decision-making of the patient-provider relationship [29, 38].

At the same time, other factors were associated with a routine discharge for all SUD patients as a cohort. Notably, uninsured patients, male patients, Black patients, and Hispanic patients also had higher hazards for routine discharge. The racial, ethnic, socioeconomic, and gender disparities of the findings raise questions about the treatment pathways when considering the entire cohort of SUD patients. That is, to what extent, if any, are these covariate associations with routine discharge related to any systemic biases, a lack of coverage, or clinician bias particularly with respect to SUD patients who did not receive a consultation [17, 39, 40]? Alternatively, mistrust in medicine, perceived discrimination, self-reliance for substance misuse healing and treatment or lack of treatment readiness may also inform these associations (e.g., for patients who may have denied outpatient treatment or patients who did not receive a consultation) [22, 40, 41].

Sample demographic differences may also warrant further investigation into patient experience as well as systemic or clinician biases. Although SUIT consultation patients were almost two years younger, their Elixhauser scores skewed higher (7.8 v. 7.4, p<0.05). Clinically, the difference in mean age may be inconsequential (48.43 v. 50.71, p<0.001), but the SUIT group’s higher mean comorbidity score likely reflects more severe infections and trauma related to misuse. The distribution of payors between groups shows disproportionate Medicaid coverage and uninsured among those who received a SUIT consultation and disproportionate Medicare coverage among non-SUIT SUD patients, while approximately one quarter of both groups had private insurance. Given that SUD cuts across socioeconomic status and that approximately 35–40% of the general population of hospitalized patients are privately insured, this distribution suggests that privately insured patients may be under-diagnosed with SUD, and uninsured and publicly-insured patients may be disproportionately diagnosed [40, 42].

The sensitivity analysis showing the SUIT consultation’s higher hazard for AMA discharge raised questions about the adequacy of SUIT treatment pathways and perhaps treatment readiness for non-treatment seeking hospitalized patients. However, when these analyses were conducted at the patient level, the direction of SUIT consultation hazard ratio reversed and was associated with a lower hazard for AMA [0.41, (0.18–0.91)]. The SUIT consultation hazard for routine discharge increased [2.95 (1.91–4.56)]. These patterns suggest that at the patient level, the service is working as intended though some perhaps uniquely different patients experience readmissions after leaving AMA. Of note, the SUIT service only operates weekdays from 9am-5pm and cannot always respond to evening and weekend consultation orders in time to alleviate withdrawal symptoms. Although a greater proportion of SUIT SUD patients discharged AMA, they likely have greater severity of SUD-related symptoms, and they are returning for care suggesting that relapse may be driving AMA discharge rather than quality of care per se.

Several study limitations should be considered. First, the observational study design lends itself to selection bias of the sample as a whole and regarding who received a SUIT consult versus who did not, which has implications for the internal validity of our results. As noted, privately insured patients may be underrepresented and underdiagnosed with SUD given their lower distribution in the sample. Though the two subgroups did not differ across race, gender, or ethnicity, patients receiving SUIT consultation were more likely to be on Medicaid or uninsured whereas the patients not receiving SUIT consultation were more likely on Medicare. SUIT patients also had a higher Elixhauser score, on average. Second, only structured data collected and entered into the EHR were available for analyses; therefore, variables such as chief complaint, misuse severity based on secondary screening scores, outpatient linkages and retention, and housing status could not be included. Future research that accounts for misuse severity as well as outpatient retention in care will help tailor treatment pathways and improve patient outcomes.

## Conclusion

SUIT consultation services have potentially impacted the outcomes of the SUD patients hospitalized at our single center in 2018 with a reduction in average LOS and a higher hazard for a routine discharge. Since this analysis, SUIT is integrating a peer recovery counseling team for patients and has strengthened relationships with community partners in order to improve the patient experience, treatment pathway, and retention in treatment. Future research would benefit from stratification by substances, particularly alcohol and opioids, in order to understand differences in LOS, 30-day readmissions, and linkages to and participation in outpatient treatment. Further investigation is needed to analyze and intervene upon potential sources of structural, institutional, and clinician biases with respect to treatment of substance misuse. These additional data may guide health systems’ intervention adaptation as well as treatment pathways beyond hospitalization that are tailored to alcohol, opioids, cocaine, and polysubstance misuse.
